# A possible coincidence of cytomegalovirus retinitis and intraocular lymphoma in a patient with systemic non-Hodgkin’s lymphoma

**DOI:** 10.1186/1743-422X-10-18

**Published:** 2013-01-07

**Authors:** Petra Svozílková, Jarmila Heissigerová, Michaela Brichová, Bohdana Kalvodová, Jan Dvořák, Eva Říhová

**Affiliations:** 1 Department of Ophthalmology, First Faculty of Medicine, Charles University in Prague and General University Hospital in Prague, U Nemocnice 2, 128 08, Prague 2, Czech Republic

**Keywords:** Cytomegalovirus, Cytomegalovirus retinitis, Foscarnet, Non-Hodgkin’s lymphoma, Rituximab, Valganciclovir

## Abstract

**Purpose:**

To present a possible coincidence of cytomegalovirus retinitis and intraocular lymphoma in a patient with systemic non-Hodgkin’s lymphoma.

**Case presentation:**

A 47-year-old woman presented with decreased visual acuity associated with white retinal lesions in both eyes. A history of pneumonia of unknown aetiology closely preceded the deterioration of vision. Five years previously the patient was diagnosed with follicular non-Hodgkin’s lymphoma. She was treated with a chemotherapy regimen comprised of cyclophosphamide, adriamycin, vincristin, and prednisone with later addition of the anti-CD20 antibody rituximab. She experienced a relapse 19 months later with involvement of the retroperitoneal lymph nodes, and commenced treatment with rituximab and ^90^Y-ibritumomab tiuxetan. A second relapse occurred 22 months after radioimmunotherapy and was treated with a combination of fludarabine, cyclophosphamide, and mitoxantrone followed by rituximab. The patient experienced no further relapses until the current presentation (April, 2010).

Pars plana vitrectomy with vitreous fluid analysis was performed in the right eye. PCR testing confirmed the presence of cytomegalovirus in the vitreous. Atypical lymphoid elements, highly suspicious of malignancy were also found on cytologic examination. Intravenous foscarnet was administered continually for three weeks, followed by oral valganciclovir given in a dose of 900 mg twice per day. In addition, the rituximab therapy continued at three monthly intervals. Nevertheless, cessation of foscarnet therapy was followed by a recurrence of retinitis on three separate occasions during a 3-month period instigating its reinduction to the treatment regime after each recurrence.

**Conclusions:**

Cytomegalovirus retinitis is an opportunistic infection found in AIDS patients as well as in bone marrow and solid organ transplant recipients being treated with systemic immunosuppressive drugs. This case presents a less common incidence of cytomegalovirus retinitis occurring in a patient with non-Hodgkin’s lymphoma. We demonstrated a possible coexistence of cytomegalovirus retinitis and intraocular lymphoma in this particular patient. The final diagnosis was based on clinical manifestations together with the course of uveitis and its response to treatment alongside the results of vitreous fluid analysis. This report highlights the importance of intraocular fluid examination in cases with nonspecific clinical manifestations. Such an examination allows for the detection of simultaneously ongoing ocular diseases of differing aetiologies and enables the prompt initiation of effective treatment.

## Background

Cytomegalovirus (CMV) retinitis is a severe sight-threatening disease which predominantly affects patients with AIDS [1-3]. CMV retinitis may also occur in patients who are lymphopenic secondary to immunosuppressive therapy after bone marrow or solid organ transplantation [4,5]. Unless effective treatment is promptly initiated, the disease may lead to progressive visual loss and blindness [6,7].

Generally, immune recovery uveitis (IRU) should be considered in the differential diagnosis of CMV retinitis. IRU is an intraocular inflammatory disorder originally described in individuals with human immunodeficiency virus (HIV) and inactive cytomegalovirus retinitis following highly active antiretroviral therapy. IRU also occurs in iatrogenically immunosuppressed individuals in the context of tapering immunosuppressive treatment [8].

This report focuses on a less common case of cytomegalovirus retinitis occurring in a patient with systemic non-Hodgkin’s lymphoma. It presents an incidence of simultaneous occurrence of cytomegalovirus retinitis and intraocular manifestation of non-Hodgkin’s lymphoma.

## Case presentation

A 47-year-old woman presented with decreased visual acuity associated with white retinal lesions in both eyes. A history of pneumonia of unknown aetiology closely preceded the deterioration of vision. Five years previously the patient was diagnosed with follicular non-Hodgkin’s lymphoma (December, 2004). She underwent eight cycles of combination chemotherapy that included cyclophosphamide, adriamycin, vincristine and prednisone with later addition of the anti-CD20 antibody rituximab. The patient was in remission for 19 months. Following a relapse with involvement of the retroperitoneal lymph nodes (February, 2007), rituximab and ^90^Y-ibritumomab tiuxetan were administered. A second relapse occurred 22 months post radioimmunotherapy (April, 2009). Four combination cycles of fludarabine, cyclophosphamide, and mitoxantrone were then undertaken leading to partial remission. Subsequent therapy included rituximab administered once per month for four months and once every third month thereafter. No further relapses were experienced from April, 2009 to April, 2010.

**Figure 1:**
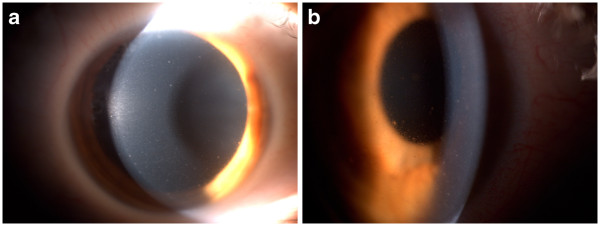
**Anterior segment of the eye. A**, large keratic precipitates on the corneal endothelium. **B,** a mild anterior chamber cellular reaction.

**Figure 2:**
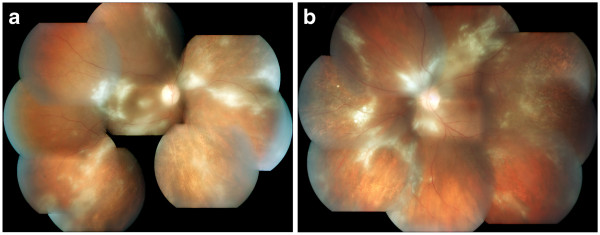
**A photograph of the fundus. A,** right eye. **B,** left eye. Apparent are retinal infiltrates and retinal necrosis, a pale optic disc, several hemorrhages in the posterior pole and white sheathing of some vessels.

At presentation (April, 2010), her best-corrected Snellen visual acuity (BCVA) was 6/12 in the right eye and 6/9 in the left eye. There were large keratic precipitates and a mild anterior chamber cellular reaction present in both eyes (Figure 1). Examination of the fundus revealed bilateral findings of moderate vitreous opacities, pale optic discs, retinal necrosis with retinal infiltrates, several hemorrhages in the posterior pole and areas of peripheral retinal atrophy. Some vessels displayed extensive white sheathing providing them with the appearance of frosted branch angiitis (Figure 2). Despite prophylactic antiviral therapy (valganciclovir 900 mg twice per day and valaciclovir 500 mg once per day) and maintenance treatment with rituximab (800 mg once per three months), the most likely diagnoses were CMV retinitis or intraocular non-Hodgkin’s lymphoma. Blood tests revealed neutropenia (0.9 × 10^9^/L) with a normal lymphocyte count of 1.2 × 10^9^/L. Serology was negative for HIV.

**Figure 3:**
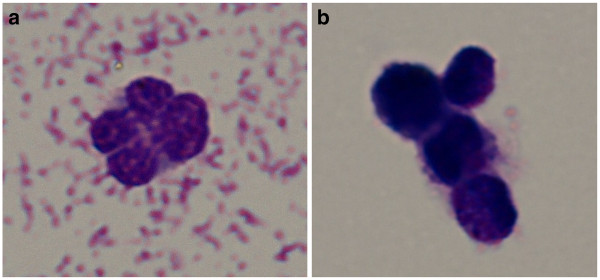
**Results of cytologic examination of vitreous fluid. A,** atypical lymphocytes highly suspected of NHL. **B,** syntitium of atypical lymphocytes in vitreous body. Microscope Olympus BX51, digital camera E-510. Objective 100x, num.ap. 1.4. May-Gruenwald-Giemsa staining.

Pars plana vitrectomy was performed in the right eye for both - diagnostic as well as therapeutic purposes (June, 2010). Vitreous fluid analysis confirmed CMV by means of PCR testing. Furthermore, cytologic examination revealed atypical lymphocytes with lobulated nuclei and basophilic cytoplasma (Figure 3) on a background of lytic cells. Two specialists confirmed the finding of highly suspicious malignant elements in the vitreous fluid. Treatment with intravenous foscarnet was given continually for three weeks followed by oral valganciclovir given in a dose of 900 mg twice per day. Rituximab therapy was also maintained and administered every three months. The diagnosis of intraocular lymphoma is usually an indication for intravitreal treatment with methotrexate in addition to systemic therapy. Intavenous methotrexate therapy was not considered in this case as PCR testing confirmed infectious uveitis of cytomegalovirus aetiology. In such infectious cases, methotrexate could probably worsen the course of ocular disease.

**Figure 4:**
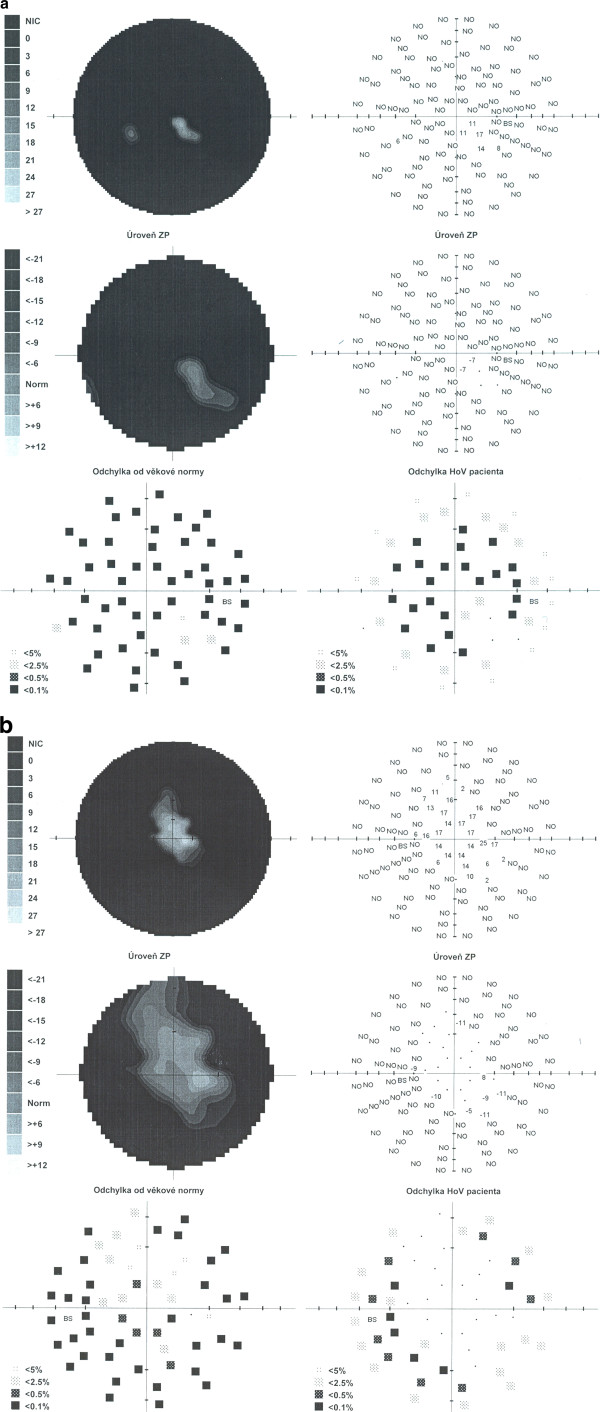
**Results of perimetry. A,** right eye. **B,** left eye. Large visual fields defects.

**Figure 5:**
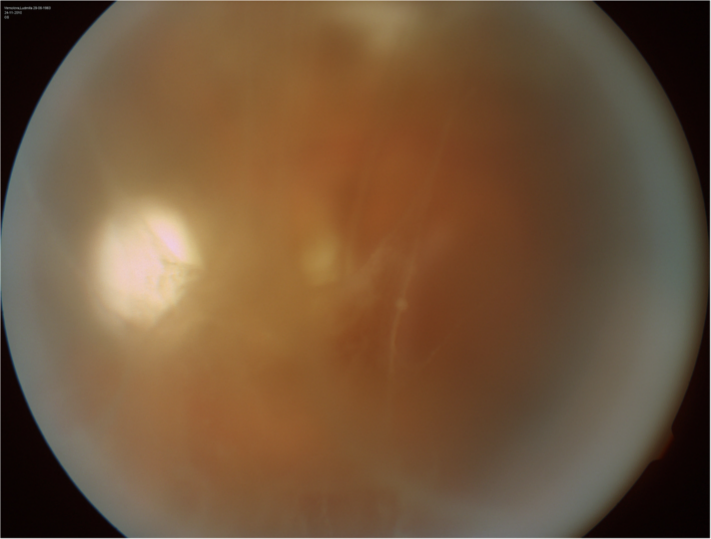
Vitreous haze in the left eye.

Retinal findings responded well to treatment and retinitis regressed. However, BCVA decreased to 6/24 in the right eye and 6/12 in the left eye, perimetry revealed scotomas (Figure 4) and moderate vitreous haze persisted in the left eye (Figure 5). The main cause of visual impairment was most likely pallor of the optic disc.

**Figure 6:**
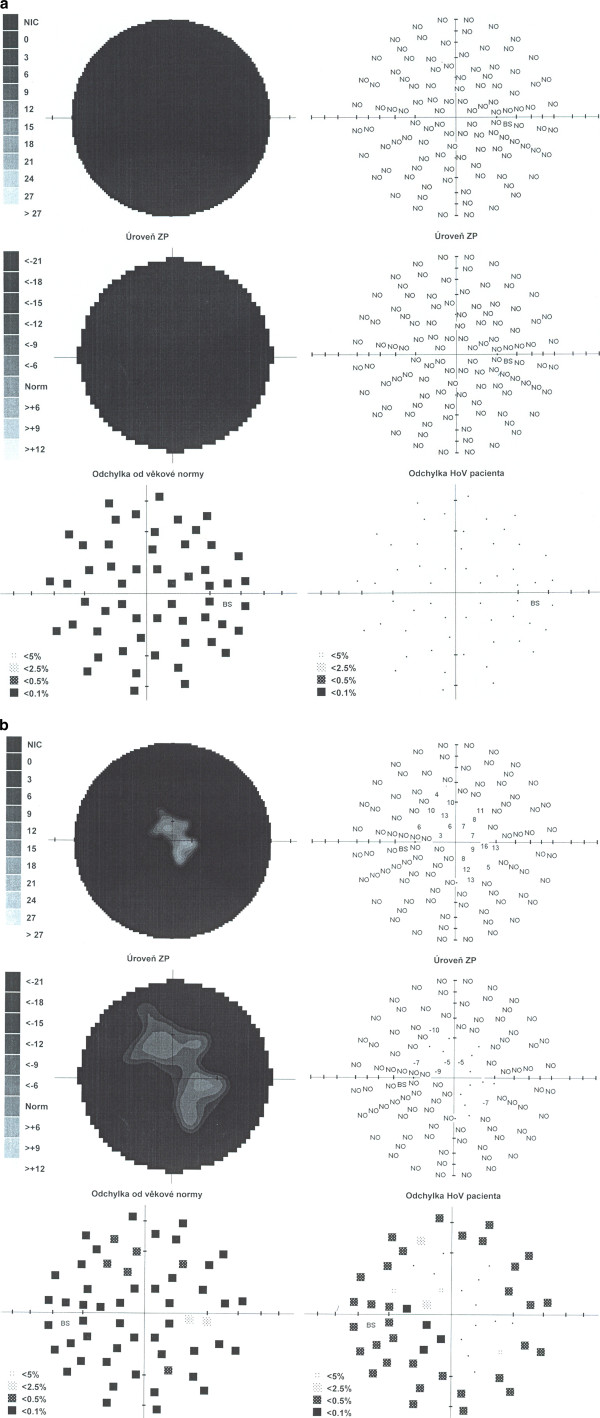
**Perimetry with progression of visual fields defects. A,** right eye. **B,** left eye.

**Figure 7:**
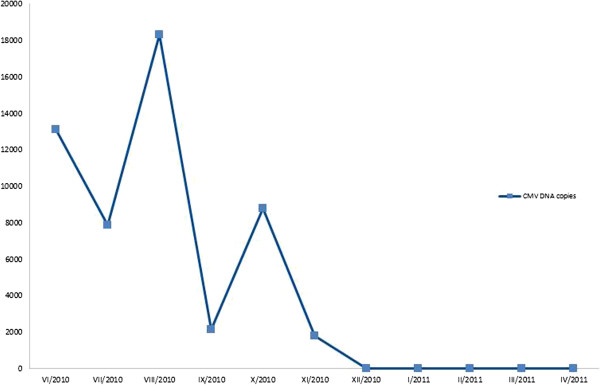
**Time course of CMV DNA copies in plasma.** Three-week long continuous intravenous foscarnet was applied in June, July and October, 2010. During September the patient was treated with foscarnet administered as a bolus three times per week.

A relapse of retinitis in the three weeks following discontinuation of foscarnet treatment necessitated its reinduction and administration once again continually for three weeks (August, 2010). BCVA reduced to hand movements in the right eye and 6/18 in the left eye and further progression of visual field defects was demonstrated (Figure 6). A further recurrence developed within one week of cessation of this therapy. Consequently, the patient was retreated with foscarnet administered as a bolus three times per week (September, 2010). This therapeutic regime proved to be insufficient as retinal lesions once again showed signs of reactivation. Moreover, an increase in plasmatic CMV DNA copies was detected (Figure 7). The patient was admitted to a hematologic clinic and treatment with intravenous foscarnet was administered continually for further three weeks (October, 2010). Systemic examination for restaging of the follicular lymphoma was also undertaken. An MRI of the brain showed no signs of lymphoma. Cerebrospinal fluid was negative for CMV. No pathological changes were revealed by immunophenotyping and cytologic examination of cerebrospinal fluid.

Retinal lesions were in regression, but plasma CMV DNA copies were still positive giving rise to consideration of cidofovir therapy. The patient declined this treatment. Maintenance therapy with rituximab was also discontinued.

Two months after the most recent foscarnet therapy (December, 2010), negativity of CMV DNA copies in the plasma was detected. However, BCVA had decreased to hand movements in the right eye and 1/60 in the left eye due to a combination of optic disc atrophy and progression of cataract.

**Figure 8:**
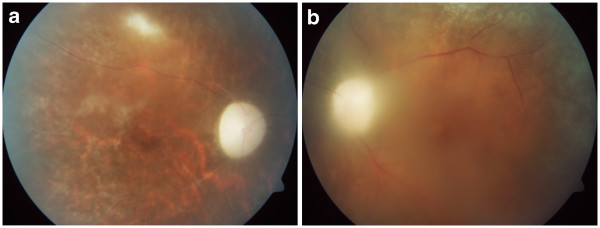
**A photograph of the fundus with retinal lesions in regression. A,** right eye. A pale optic disc and fibrosis of some vessels. **B,** left eye. A pale optic disc and persisting moderate vitreous haze.

Patient follow-up was scheduled for every 8-10 weeks as retinal lesions were inactive (Figure 8). Nevertheless, moderate vitreous haze remained in the left eye resembling that which occurs in patients with intraocular lymphoma. It is therefore believed that both CMV retinitis and intraocular lymphoma contributed to the intraocular findings. This opinion was strongly supported in the results of vitreous fluid analysis of the right eye. Undertaking diagnostic-therapeutic pars plana vitrectomy in the left eye imposed a high risk and the patient declined this surgical procedure.

In August 2011, trepanobiopsy confirmed a transformation of follicular lymphoma into secondary acute myeloid leukemia. Palliative treatment was indicated for this disease.

At the last follow-up in March 2012, no visual acuity was recordable in the right eye (blind eye) and hand movements in the left eye. Bilateral progression of cataract prevented good fundal views although some white retinal lesions were apparent.

The patient died in another hospital in June 2012 where unfortunately no post-mortem examination was performed.

## Discussion

Cytomegalovirus retinitis commonly presents in immunocompromised lymphocytopenic patients, mainly in patients with AIDS. Occurances of CMV retinitis in immunocompetent patients although rare, have been described [9].

This report presents a case of bilateral CMV retinitis in an HIV negative patient with non-Hodgkin’s lymphoma who had a normal lymphocyte count. Intraocular inflammation of CMV aetiology occurred despite oral prophylaxis with valganciclovir. Similar cases have been published, where valganciclovir prophylaxis or ganciclovir/valganciclovir treatment were unable to prevent CMV retinitis because of drug resistance [10,11].

It is critical to differentiate lymphomatous chorioretinal infiltration from opportunistic CMV infection in order to obtain an accurate diagnosis and initiate effective treatment in patients with non-Hodgkin’s lymphoma. It is well known that the differential diagnosis of such cases should also include fulminant toxoplasmic chorioretinitis, mycotic endophthalmitis, tuberculosis, syphilis, herpes simplex or varicella zoster retinitis. Where clinical findings are not specific, intraocular fluid/tissue analysis may be necessary. Derzko-Dzulynsky *et al*. reported the case of a patient with follicular non-Hodgkin’s lymphoma, who developed a chorioretinal infiltrate that was initially thought to represent an intraocular manifestation of malignant disease. The patient received radiation treatment appropriate for intraocular lymphoma. The lesion progressed further and after re-evaluation, which included vitreous fluid examination, a diagnosis of cytomegalovirus retinitis was made and therapy initiated [12]. Another case of a patient with follicular non-Hodgkin’s lymphoma and retinal infiltrate was described by Gooi *et al*. In this patient, CMV retinitis mimicked intraocular lymphoma and a retinal biopsy was required for assessment of the final diagnosis [13].

In the case presented here, an initial diagnostic dilemma was caused by the clinical appearance of lesions and subsequent results of vitreous fluid analysis - both of which provided evidence of CMV retinitis and concurrent intraocular lymphoma. To date, it appears that similar cases of two coexisting ocular diseases of differing aetiologies both in an active course – malignant masquerade syndrome and infectious uveitis, have not been reported. Thus, this patient was treated by means of intravenous foscarnet applied continually. However, management of non-Hodgkin’s lymphoma involved only maintenance treatment with rituximab applied every three months because of a negative restaging examination.

A therapeutic regime using foscarnet administration as a bolus three times per week was ineffective and led to a relapse of retinitis. Hence, continual intravenous application was necessary to control CMV retinitis activity. On the other hand, a study performed in patients with HIV infection showed that although foscarnet was given as a continuous intravenous infusion, there was a large variation in foscarnet concentration in the plasma of patients [14].

A suspicion remained that the persistent vitreous haze in the left eye, atypical at this degree for CMV retinitis, was an intraocular manifestation of non-Hodgkin’s lymphoma. This could not be confirmed as the patient declined vitreous fluid analysis.

## Conclusions

This case reports a possible coincidence of cytomegalovirus retinitis and intraocular lymphoma in a patient with systemic non-Hodgkin’s lymphoma. The final diagnosis was based on clinical manifestations, the course of uveitis and its response to treatment as well as the results of vitreous fluid analysis. This report highlights the importance of intraocular fluid examination, especially in cases with nonspecific clinical manifestations. Such an examination allows for detection of simultaneous ongoing ocular diseases of two different aetiologies and thus enables the prompt initiation of effective treatment.

## Consent

Written informed consent was obtained from the patient for publication of this case report and any accompanying images. A copy of the written consent is available for review by the Editor-in-Chief of this journal.

## Competing interests

The authors declare that they have no competing interests.

## Authors’ contributions

PS, JH, MB and ER carried out the ophthalmologic examination of the patient. All of these authors participated in management of the therapeutic approach. BK and JD performed the surgical procedure, pars plana vitrectomy. All authors read and approved the final manuscript.
